# Comparison of registered and published outcomes in randomized controlled trials: a systematic review

**DOI:** 10.1186/s12916-015-0520-3

**Published:** 2015-11-18

**Authors:** Christopher W. Jones, Lukas G. Keil, Wesley C. Holland, Melissa C. Caughey, Timothy F. Platts-Mills

**Affiliations:** Department of Emergency Medicine, Cooper Medical School of Rowan University, One Cooper Plaza, Suite 152, Camden, NJ 08103 USA; School of Medicine, University of North Carolina Chapel Hill, 321 S Columbia St, Chapel Hill, NC 27516 USA; Department of Biology, University of North Carolina Chapel Hill, Coker Hall, 120 South Rd, Chapel Hill, NC 27599 USA; Department of Medicine, University of North Carolina Chapel Hill, 125 MacNider Hall, CB 7005, Chapel Hill, NC 27599 USA; Department of Emergency Medicine, University of North Carolina Chapel Hill, 170 Manning Dr. CB#7594, Chapel Hill, NC 27599 USA

**Keywords:** Clinicaltrials.gov, Primary outcome, Publication bias, Reporting bias, Selection bias, Trial registration

## Abstract

**Background:**

Clinical trial registries can improve the validity of trial results by facilitating comparisons between prospectively planned and reported outcomes. Previous reports on the frequency of planned and reported outcome inconsistencies have reported widely discrepant results. It is unknown whether these discrepancies are due to differences between the included trials, or to methodological differences between studies. We aimed to systematically review the prevalence and nature of discrepancies between registered and published outcomes among clinical trials.

**Methods:**

We searched MEDLINE via PubMed, EMBASE, and CINAHL, and checked references of included publications to identify studies that compared trial outcomes as documented in a publicly accessible clinical trials registry with published trial outcomes. Two authors independently selected eligible studies and performed data extraction. We present summary data rather than pooled analyses owing to methodological heterogeneity among the included studies.

**Results:**

Twenty-seven studies were eligible for inclusion. The overall risk of bias among included studies was moderate to high. These studies assessed outcome agreement for a median of 65 individual trials (interquartile range [IQR] 25–110). The median proportion of trials with an identified discrepancy between the registered and published primary outcome was 31 %; substantial variability in the prevalence of these primary outcome discrepancies was observed among the included studies (range 0 % (0/66) to 100 % (1/1), IQR 17–45 %). We found less variability within the subset of studies that assessed the agreement between prospectively registered outcomes and published outcomes, among which the median observed discrepancy rate was 41 % (range 30 % (13/43) to 100 % (1/1), IQR 33–48 %). The nature of observed primary outcome discrepancies also varied substantially between included studies. Among the studies providing detailed descriptions of these outcome discrepancies, a median of 13 % of trials introduced a new, unregistered outcome in the published manuscript (IQR 5–16 %).

**Conclusions:**

Discrepancies between registered and published outcomes of clinical trials are common regardless of funding mechanism or the journals in which they are published. Consistent reporting of prospectively defined outcomes and consistent utilization of registry data during the peer review process may improve the validity of clinical trial publications.

**Electronic supplementary material:**

The online version of this article (doi:10.1186/s12916-015-0520-3) contains supplementary material, which is available to authorized users.

## Background

The designation of a clearly defined, pre-specified primary outcome is a critically important component of a clinical trial [1]. Inconsistencies between planned and reported outcomes threaten the validity of trials by increasing the likelihood that chance or selective reporting, rather than true differences between treatment and control groups, account for primary outcome differences as reported at the time of publication. These inconsistencies can have direct consequences for physician decision-making and policies that influence patient care [2].

ClinicalTrials.gov was established in 2000, in large part to encourage consistency and transparency in the reporting of clinical trial outcomes. Since 2005 the International Committee of Medical Journal Editors (ICMJE) has explicitly supported this goal by mandating the registration of clinical trials in a publicly available registry prior to beginning trial enrollment as a condition of publication in member journals [3]. Additionally, both the United States and the European Union have passed legislation requiring the prospective registration of most clinical trials involving drugs or devices [4, 5]. As a result of these policies, trial registration has dramatically increased [6], and registries are now important tools for assessing consistency between planned and published primary outcomes.

Reported discrepancy rates between registered clinical trial outcomes and published outcomes range from less than 10 % of trials to greater than 60 % [7–9]. It is unknown whether this wide range in results stems from differences in characteristics of the included trials or from differences in how investigators assessed outcome consistency. It is also unclear which estimates are most representative of clinical trials in general. A better understanding of the problem of outcome inconsistencies may help define the frequency and impact of inappropriate changes to trial outcomes and identify subgroups of clinical trials that warrant further attention. Additionally, this knowledge may help inform future regulations aimed at improving trial transparency. We conducted a systematic review of published comparisons of registered and published primary trial outcomes in order to provide a comprehensive assessment of the observed prevalence and nature of outcome inconsistencies.

## Methods

### Search strategy and study selection

We performed a search for studies that evaluated the agreement between primary trial outcomes as documented in clinical trials registries and as described in published manuscripts. Studies were eligible if they included data from clinical trials, utilized either ClinicalTrials.gov or any registry meeting World Health Organization (WHO) Registry Criteria (Version 2.1), and included comparisons to outcomes as defined in published manuscripts. We excluded studies published prior to 2005, because our objective was to characterize inconsistencies between trial registries and publications, and registries were used infrequently prior to the 2005 ICMJE statement requiring trial registration. Manuscripts were also excluded if they were secondary reports of studies for which the relevant findings had previously been published, or if they were published in a language other than English. Reports of individual trials, case reports, and editorials were excluded. We searched MEDLINE via PubMed and CINAHL in August 2014, and EMBASE in September 2014. Full details from each search are available in Additional file 1: Appendix A. Additionally, we reviewed the citation lists from each included manuscript for additional eligible studies.

Two investigators (LGK, WCH) independently screened the titles and abstracts of manuscripts identified through the database searches to identify potentially eligible studies for further assessment. Discrepancies were resolved by a third investigator (CWJ). Following this step, two investigators then independently assessed each remaining full-length manuscript for inclusion in the study. A third investigator again reviewed all discrepancies, and the final list of included studies was determined by group consensus.

The primary outcome of interest for this systematic review was the proportion of trials in which the registered and published primary outcomes were different. Secondary outcomes were differences between registered and published secondary outcomes and the relationship between registered and published outcomes on the statistical significance of published outcomes.

### Data extraction

Two investigators independently performed data extraction for each eligible study using a standardized data form. For each study, investigators recorded the total number of trials examined and the number of trials for which a discrepancy was present between the registered and published primary outcome. WHO-approved trial registries must save a history of changes made to registry records, and we also recorded whether study authors reported reviewing these changes. When included studies utilized this feature to report registered outcomes at various time points, we used the primary outcome registered at the time enrollment started as the registered outcome [3]. Additional data fields included the nature of primary outcome discrepancies, categorized according to a modified version of the approach developed by Chan et al. [10] These categories are: a registered primary outcome was omitted in the manuscript; an unregistered primary outcome was introduced in the manuscript; a registered primary outcome was described as a secondary outcome in the manuscript; a published primary outcome was described as a secondary outcome in the registry; and the time of primary outcome assessment differed between the registry and manuscript. When possible, the data abstractors also recorded information about the publishing journals or clinical specialties for trials included in each study, publication year of included trials, registry databases utilized for each study, the timing of trial registration with respect to participant enrollment, and the proportion of outcome discrepancies favoring statistical significance in a published manuscript.

### Data synthesis

Data were analyzed in accordance with the methodology recommended in the Cochrane Handbook for Systematic Reviews of Interventions, where relevant, and results are reported in accordance with the Preferred Reporting Items for Systematic Reviews and Meta-Analyses (PRISMA) statement (Additional file 2: Appendix B) [11, 12]. For each included study we calculated the proportion of trials with primary outcome discrepancies, along with 95 % confidence intervals. Significant heterogeneity with respect to the methods of the included studies and substantial statistical heterogeneity prevented us from performing pooled analyses. Pre-specified analyses included the assessment of primary outcome discrepancies among prospectively registered trials, secondary outcome discrepancies, and the proportion of outcome discrepancies favoring statistical significance in a published manuscript. Following our initial data analysis, we decided to compare primary outcome discrepancy rates between studies in which authors generated a list of published trials and searched for corresponding registry entries with studies in which a list of registered trials was used as the basis for a publication search, because both strategies were common among the included studies.

We assessed the risk of bias of included studies using a modified version of the Newcastle-Ottawa Scale for assessing the quality of cohort studies, in which the included studies were assessed based on the following factors: representativeness of the included cohort; ascertainment of the link between trial registry and manuscript; method of assessing primary outcome agreement; enrollment window of included trials; and the analysis of prospectively registered outcomes (Additional file 3: Appendix C) [13]. We used the Wilson score method with continuity correction to calculate 95 % confidence intervals [14]. Analyses were conducted using Stata 14 (College Station, TX, USA).

## Results

### Description of included studies

**Fig. 1:**
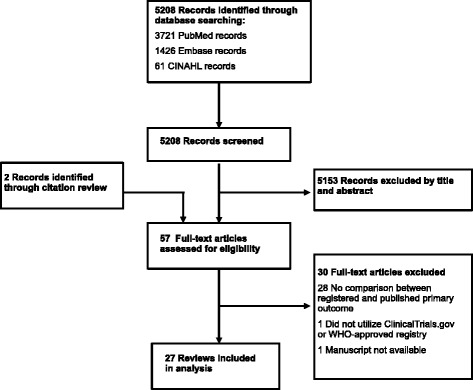
Flowchart of study selection process

Database searches identified 5208 records. Following exclusions based on title and abstract review, we assessed 57 full manuscripts for eligibility. Twenty-five studies identified by the database search and another two studies identified through the citation review met inclusion criteria for a total of 27 eligible studies that compared registered and published primary outcomes (Fig. 1) [7–9, 15–38]. Characteristics of these studies, including publication dates, a description of included trials, and search methods, are reported in Table 1. Detailed ratings regarding risk of bias for the included studies are provided in Additional file 3: Appendix C. Risk of bias was moderate to high for most studies; five studies were judged to be at low risk of bias [7, 16, 20, 24, 30].

**Table 1:** Characteristics of included studies

Authors	Journal and publication year	Publication dates of included trials	Trials registries used	Description of included trials	Trials included in outcome consistency assessment	Date of initial registry search	Method of identifying included studies	Used prospectively registered outcomes in discrepancy analysis	Method of characterizing outcome discrepancies	Subgroup analyses	Additional comparisons between registry and manuscript
Anand et al. [16]	Intensive Care Med. 2014	2005–2011	ClinicalTrials.gov, ISRCTN, anzctr.org.au	Critical care trials	55	Unknown	Publication search	Yes	Not applicable	Journals following ICMJE recommendations	Sample size
Bourgeois et al. [17]	Ann Intern Med. 2010	Not available	ClinicalTrials.gov	Trials of anticholestermics, antidepressants, antipsychotics, proton pump inhibitors, vasodilators	85	31 August 2009	Registry search	No	Not applicable	Funding source	None
Chahal et al. [18]	Am J Sports Med. 2012	Not available	ClinicalTrials.gov	Orthopedic sports medicine trials	20	Unknown	Registry search	No	Modified version of Chan’s classification	None	Sample size, inclusion criteria
Ewart et al. [15]	Ann Fam Med. 2009	2006–2007	ClinicalTrials.gov, ISRCTN, anzctr.org.au, national register for included trials	Trials in high-impact journals	110	Unknown	Publication search	No	Chan	None	None
Gandhi et al. [19]	BMC Musculoskelet Disord. 2011	1999–2010	ClinicalTrials.gov	Trials related to orthopedic trauma	20	July 2010	Registry search	No	Chan	Funding source	Sample size
Hannink et al. [20]	Ann Surg. 2013	2007–2012	ClinicalTrials.gov, ISRCTN, anzctr.org.au, “Other”	Trials assessing surgical interventions	152	Unknown	Publication search	Yes	Chan	General medical vs surgical journals	None
Hartung et al. [21]	Ann Intern Med. 2014	Not available	ClinicalTrials.gov	Random sample of phase 3 or 4 trials completed before January 2009 with results available in ClinicalTrials.gov	110	15 February 2011	Registry search	No	Not applicable	None	Results, adverse events
Huić et al. [22]	PLoS One. 2011	2005–2005	ClinicalTrials.gov	Trials in ICMJE member journals	152	Unknown	Publication search	Yes	Chan	None	Title, start date, trial methods, sample size
Hutfless et al. [23]	Abstract from American Gastroenterological Association 2013 meeting	Not available	ClinicalTrials.gov	Trials of Crohn’s disease medications	25	July 2010	Unknown	No	Not applicable	None	None
Jones et al. [24]	Ann Emerg Med. 2012	2008–2011	ClinicalTrials.gov, ISRCTN, WHO portal, national register for included trials	Trials published in emergency medicine journals	57	Unknown	Publication search	Yes	Chan	None	None
Khan et al. [25]	Arthritis Rheum. 2012	2002–2007	ClinicalTrials.gov	Trials of drug therapy for rheumatoid arthritis	6	Unknown	Publication search	No	Not applicable	None	None
Killeen et al. [26]	Ann Surg. 2014	2009–2010	ClinicalTrials.gov, ISRCTN, WHO portal, national register for included trials	Trials from surgical journals	108	Unknown	Publication search	No	Chan	None	None
Li et al. [27]	Scand J Gastroenterol. 2013	2009–2012	“Registries accepted by the ICMJE”	Gastroenterology trials	155	Unknown	Publication search	No	Chan	General medical vs gastroenterology & hepatology journals	None
Liu et al. [28]	BMJ Open. 2013	Not available	WHO registry portal plus Korean Clinical Research Information Service	Trials of traditional Chinese medicine	65	July 2012	Registry search	No	Not applicable	None	Sample size, study methods
Mathieu et al. [7]	JAMA. 2009	2008	ClinicalTrials.gov, ISRCTN, WHO portal, national register for included trials	Cardiology, rheumatology, and gastroenterology trials in high-impact general medical and specialty journals	147	Unknown	Publication search	Yes	Chan	General medical vs specialty journals	None
Mathieu et al. [29]	Joint Bone Spine. 2012	2006–2008	ClinicalTrials.gov, ISRCTN	Trials on rheumatoid arthritis, osteoarthritis, and spondyloarthropathies	40	Unknown	Publication search	No	Not applicable	None	None
Milette et al. [30]	J Psychosom Res. 2011	2008–2009	ClinicalTrials.gov, ISRCTN, WHO portal, national register for included trials	Trials in psychosomatic and behavioral health journals	1	Unknown	Publication search	Yes	Not described	None	None
Nankervis et al. [31]	J Invest Dermatol. 2012	2007–2011	WHO portal	Eczema trials from the Global Resource for Eczema Trials (GREAT) database	8	Unknown	Publication search	Yes	Differences in primary and published outcomes or outcome timing	None	None
Pinto et al. [32]	Phys Ther. 2013	2009	ClinicalTrials.gov, ISRCTN, WHO portal, anzctr.org.au, national register for included trials	Physical therapy trials indexed in the Physiotherapy Evidence Database (PEDro)	62	Unknown	Publication search	No	Chan	None	None
Rosenthal et al. [33]	Ann Surg. 2013	2010	ClinicalTrials.gov, ISRCTN, WHO portal	Trials from high impact general surgical journals	51	Unknown	Publication search	No	Chan	Funding source, registration timing	Trial methods, sample size, analytical plan
Ross et al. [8]	PLoS Med. 2009	Not available	ClinicalTrials.gov	Random sample of phase 2–4 trials registered in ClinicalTrials.gov and completed by June 2007	198	Unknown	Registry search	No	Not applicable	None	None
Smith et al. [34]	J Arthroplasty. 2012	2006–2011	ClinicalTrials.gov	Arthroplasty trials	25	July 2010	Registry search	No	Chan	Funding source	Sample size
Smith et al. [9]	Pain. 2013	2002–2011	ClinicalTrials.gov	Trials from the Repository of Registered Analgesic Clinical Trials	79	1 December 2011	Registry search	No	Chan	Funding source, trial phase	Analytical plan, missing data plan
Vera-Badillo et al. [35]	Ann Oncol. 2013	1995–2011	ClinicalTrials.gov	Breast cancer trials	30	Unknown	Publication search	No	Not applicable	Funding source	None
Walker et al. [36]	JRSM Open. 2014	2011–2012	ClinicalTrials.gov, ISRCTN, national register based on first author nationality	Trials from the Journal of the American Medical Association and BMJ	75	Unknown	Publication search	Yes	Chan	None	Sample size, analytical plan
Wildt et al. [37]	BMJ Open. 2011	1980–2011	ClinicalTrials.gov	Trials on diseases of the digestive system	66	1 January 2009	Registry search	No	Not described	None	Sample size
You et al. [38]	J Clin Oncol. 2012	2005–2009	ClinicalTrials.gov, ISRCTN	Adult solid tumor oncology trials	134	August 2009	Publication search	No	Not applicable	Funding source, journal impact factor, publication year, location of author	None

*ISRCTN* International Standard Randomised Controlled Trial Number registry, *ICMJE* International Committee of Medical Journal Editors, *WHO* World Health Organization

**Fig. 2:**
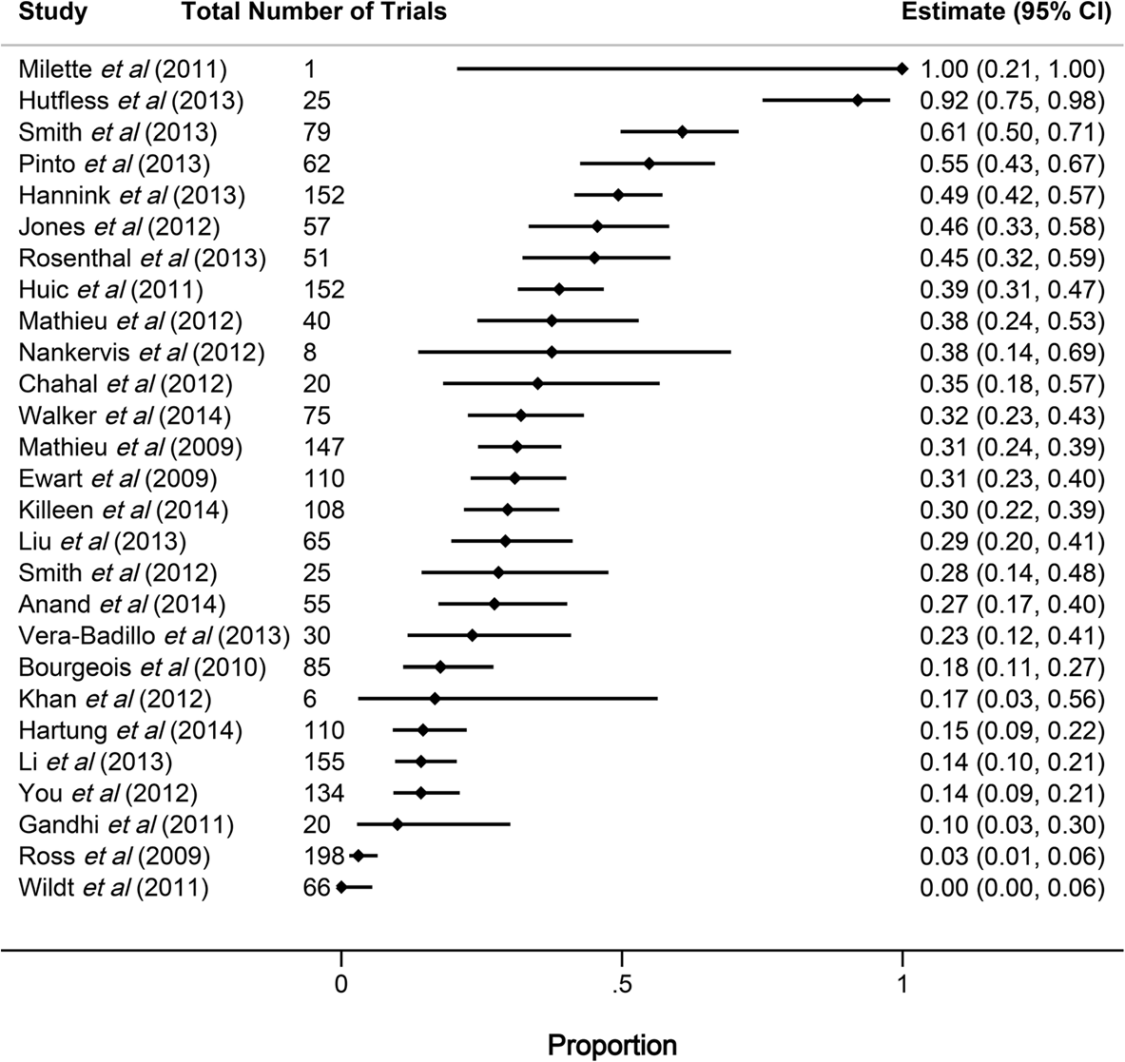
Forest plot showing the proportion of registered and published primary outcome discrepancies among included studies. Horizontal bars indicate 95 % confidence intervals (*CI*) for proportions

Among the included studies, the median number of trials assessed for outcome discrepancies was 65 (interquartile range [IQR] 25–110), and the median proportion of studies for which a discrepancy was identified between the registered and published primary outcome was 31 %, though this varied substantially across the included studies (range 0 % [0/66] to 100 % [1/1], IQR 17–45 %; Fig. 2).

Sixteen studies provided detailed information about the nature of observed outcome discrepancies (Table 2). Among these studies, discrepancies fitting each of the five categories described by Chan et al. were common [10]: for five studies the most common discrepancy was omission of a registered primary outcome [15, 19, 20, 30, 34]; for four studies the most common discrepancy was publication of an unregistered primary outcome [7, 22, 31, 36]; one study reported equal numbers of these two types of discrepancies [26]; for three studies the most commonly observed discrepancy was reporting the registered primary outcome as a secondary outcome [18, 24, 27]; and for three studies the most common discrepancy was a change in the timing of primary outcome assessment [9, 32, 33].

**Table 2:** Nature of reported primary outcome discrepancies

Study	Included trials; N	Published articles with new primary outcome; N (%)	Registered primary outcome not reported in published article; N (%)	Registered primary outcome reported as secondary in published article; N (%)	Published primary outcome described as secondary in registry; N (%)	Timing of primary outcome assessment differs between registry and article; N (%)
Chahal et al., 2012 [18]	20	3 (15)		4 (20)		
Ewart et al., 2009 [15]	110	10 (9)	20 (18)	6 (5)	3 (3)	
Gandhi et al., 2011 [19]	20	0 (0)	2 (10)	0 (0)	0 (0)	
Hannink et al., 2013 [20]	152	24 (16)	32 (21)	8 (5)	14 (9)	9 (6)
Huić et al., 2011 [22]	152	49 (32)	2 (1)	7 (5)	1 (1)	
Jones et al., 2012 [24]	57	9 (16)	3 (5)	10 (18)	9 (16)	5 (9)
Killeen et al., 2014 [26]	108	9 (8)	9 (8)	6 (6)	6 (6)	7 (6)
Li et al., 2013 [27]	155	7 (5)	5 (3)	9 (6)	6 (4)	6 (4)
Mathieu et al., 2009 [7]	147	22 (15)	15 (10)	6 (4)	8 (5)	4 (3)
Milette K et al., 2011 [30]	1	0 (0)	1 (100)	0 (0)	0 (0)	0 (0)
Nankervis et al., 2012 [31]	8	2 (25)				1 (13)
Pinto et al., 2013 [32]	62	12 (19)	8 (13)	17 (27)	6 (10)	18 (29)
Rosenthal et al., 2013 [33]	51	10 (20)	9 (18)	11 (22)	7 (14)	12 (24)
Smith et al., 2012 [34]	25	2 (8)	3 (12)	2 (8)		
Smith et al., 2013 [9]	79	4 (5)	5 (6)	6 (8)	2 (3)	38 (48)
Walker et al., 2014 [36]	75	10 (13)	2 (3)	2 (3)	1 (1)	5 (7)
Wildt et al., 2011 [37]	66	0 (0)	0 (0)	0 (0)	0 (0)	0 (0)
Totals	1288	173 (13)	116 (9)	94 (7)	63 (5)	105 (8)

Not all studies provided data for each possible category

### Subgroup analyses within included studies

Nine studies compared registered and published primary outcomes for trials that received industry funding and those that did not. One study observed that industry sponsored trials were significantly less likely to have outcome inconsistencies than were trials with other funding sources [17]. The remaining eight studies observed no significant relationship between funding source and outcome consistency [9, 15, 16, 24, 33–35, 38].

The authors of three studies compared primary outcome consistency between general medical journals and specialty journals. Of these, one study compared trials published in general medical journals to those published in specialty surgical journals, finding a higher rate of outcome inconsistencies among trials published in the surgical journals [20]. Another study compared trials published in general medical journals to those in gastroenterology journals, and the remaining study compared trials from general medical journals with those published in cardiology, rheumatology, or gastroenterology specialty journals. Neither of the latter two studies observed a difference in discrepancy rate according to journal type [7, 27].

**Fig. 3:**
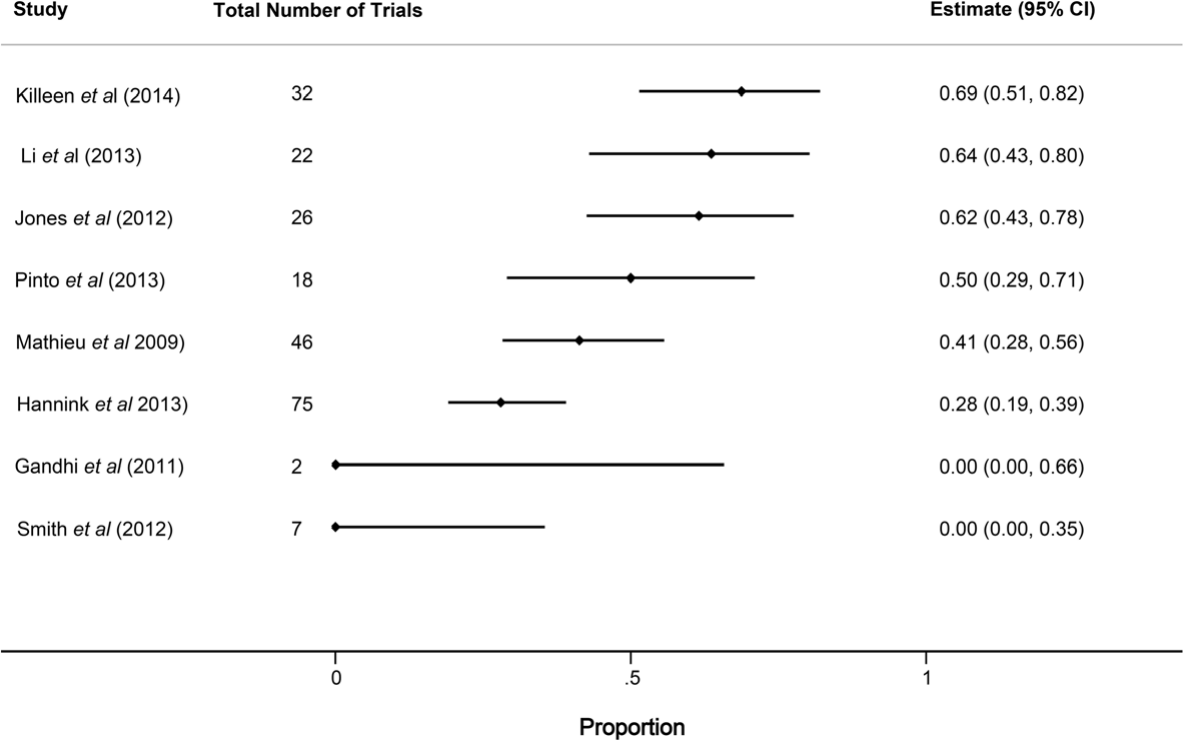
Forest plot showing the proportion of trials with primary outcome changes favoring statistically significant published outcomes. Horizontal bars indicate 95 % confidence intervals (*CI*) for proportions

Eight studies analyzed the impact that changes to registered primary outcomes had on the statistical significance of published primary outcomes for included trials (Fig. 3); an additional study reported the impact of an outcome change for a single trial, finding this change to favor statistical significance of the published outcome [30]. When it could be assessed, outcome changes frequently favored the publication of statistically favorable results (median 50 %, IQR 28–64 %). However, in as many as half of the included trials, the impact of outcome changes on the favorability of the published results could not be assessed [7]. Consequently, these data likely underestimate the true impact of outcome changes on the reported favorability of published results.

### Heterogeneity among studies

We performed several additional analyses in order to explore the substantial variability in reported outcome agreement between included studies. Of the five studies judged to be at low risk of bias, one included an outcome comparison from just a single trial, finding the registered and published outcomes to be discrepant [30]. The other four studies identified outcome discrepancies in between 27 % (15/55) and 49 % (75/152) of trials [7, 16, 20, 24]. The 22 studies determined to be at moderate to high risk of bias showed less consistency in their reported outcome discrepancies, ranging from 0 % (0/66) to 92 % (23/25), with a median discrepancy rate of 29 % (IQR 14–38 %).

Only one study explicitly limited inclusion to trials completed after the 2005 deadline for registration established by the ICMJE [16], and none limited inclusion to trials completed after the 2007 U.S. Food and Drug Administration Amendment Act expanded registration requirements and authorized civil fines for unregistered trials. However, eight studies limited inclusion to trials *published* after 2007 [7, 24, 26, 27, 30, 32, 33, 36]. Among these eight studies, the incidence of primary outcome changes between registry entries and manuscripts was similar to the findings observed among all included studies, with a median discrepancy rate of 38 % (IQR 30–50 %).

**Fig. 4:**
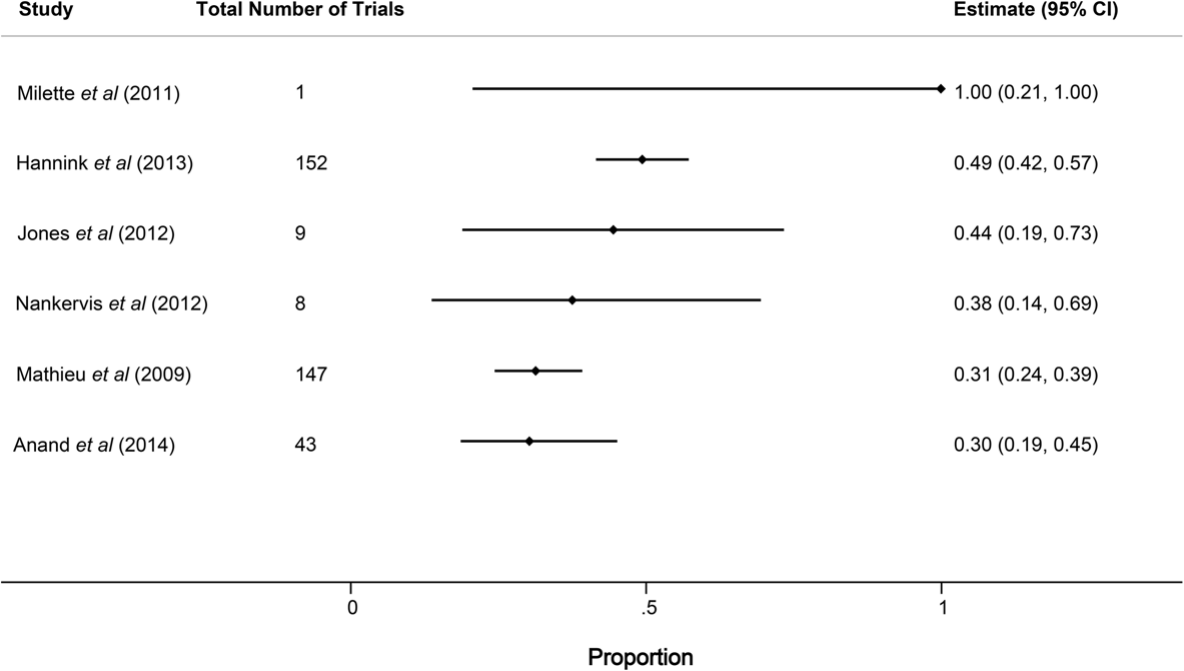
Forest plot showing the proportion of primary outcome discrepancies among prospectively registered trials. Horizontal bars indicate 95 % confidence intervals (*CI*) for proportions

Eight of the included studies examined the history of changes made to each registry record in order to compare published outcomes with the primary outcome as stated in the registry at either the time of study initiation or prior to completion of enrollment [7, 16, 20, 22, 24, 30, 31, 36]. In six of these cases, outcome discrepancy data were available specifically for trials that had been prospectively registered. Among these studies, the median proportion of prospectively registered studies with discrepancies between the registry and published manuscript was 41 % (range 30 % [13/43] to 100 % [1/1], IQR 33–48 %), and the lowest reported discrepancy rate was 30 % (Fig. 4). One study, published only in abstract form, used the ClinicalTrials.gov history of changes feature to identify trials with consistent outcomes at each of three time points (registry entry at time of initial registration, publication, current registry entry as of July 2010) and found discrepancies in 23 of 25 randomized controlled trials (92 %) [23]. The remaining 18 studies compared the current registry entry with the published outcome and did not take into account registry changes over time; among these the median primary outcome discrepancy rate was 26 % (IQR 14–34 %). The higher observed discrepancy rate among studies that exclusively utilized prospectively defined outcomes suggests that registered outcomes are often retrospectively changed to match published outcomes. This observation also likely contributes to heterogeneity in the reported results among the studies included in this analysis.

In order to match registry data with published manuscripts, 17 studies began with a cohort of published trials and searched trial registries to identify registry entries corresponding to these published manuscripts. Among these studies, the rate of unregistered trials ranged from 1 % to 94 % (median 38 %, IQR 20–71 %). Among the six studies that reported detailed results on the timing of randomized controlled trial registration, compliance with the ICMJE policy requiring prospective trial registration was poor [16, 22, 24, 27, 32, 33]. The median observed rate of prospective registration in these six studies was 15 % (range 9–31 %). Nine studies began with a group of registered trials and searched for corresponding publications [8, 9, 17–19, 21, 28, 34, 37], and one study did not specify which method was used [23]. The median percentage of primary outcome discrepancies among the 17 studies in which authors generated a list of published trials and searched for corresponding registry entries was 32 % (IQR 25–45 %), as compared to 18 % (IQR 6–32 %) among the nine studies in which a list of registered trials was used as the basis for a publication search.

### Additional analyses

The authors of the included studies observed high rates of ambiguity in the way primary outcomes were defined in registry entries. Eighteen of the 27 studies reported the number of included registry entries in which primary outcomes were undefined or unclear, with rates of ambiguously defined outcomes ranging from 4 % to 48 % (median 15 %, IQR 7–27 %) [7–9, 15, 17, 20, 22, 24, 26, 28–32, 34, 36–38]. In many cases this ambiguity was noted to limit assessments of outcome consistency. Inconsistencies between registered and published secondary outcomes were also common. Ten studies evaluated the consistency of secondary outcomes; the median rate of observed secondary outcome discrepancies among these studies was 54 % (IQR 33–68 %) [15, 18, 19, 21, 22, 28, 32–34, 37].

## Discussion

We identified 27 studies that compared registered and published trial outcomes. Among these studies, the proportion of discrepant primary outcomes between registration and publication was highly variable, with four studies observing outcome changes in more than 50 % of trials, and eight studies observing outcomes changes in fewer than 20 %. The frequency with which discrepancies were identified did not appear to vary based on funding source, journal type, or when trials were published, though our ability to detect potential differences was limited by relatively small sample sizes and unstandardized methods across the included studies. Most of the studies identified were similar in regard to the time frame analyzed and the journals and clinical topics studied. Additionally, nearly all of the included studies used a consistent method of categorizing outcome differences, based on the scheme established by Chan et al. [10]. One important source of the variability in reported results is that most studies compared published outcomes to the outcome listed in the registry at the time of the search, without attempting to identify the outcome registered at the inception of the study. For the six studies that compared published outcomes with the outcomes registered prior to the initiation of patient enrollment, the frequency of discrepancies were more similar to one another and higher than for the remaining 21 studies. Because the comparison with the outcome prior to patient enrollment is the most relevant for those attempting to understand the impact of outcome switching on the validity of results of clinical trials, the median value from these six studies (41 %, IQR 33–48 %) provides an informative estimate for characterizing the extent of this problem.

This finding supports previous observations that registry entries are often either initiated or modified after trial completion [7, 22]. Additionally, it suggests that for surveillance studies of registry practices to be meaningful and reproducible, authors should utilize best practices to improve study validity. These include assessing changes to registry entries over time, establishing clear definitions for discrepant outcomes, and utilizing multiple independent raters to compare registered and published outcomes.

While clinical trial registries and recent legislative efforts mandating their use have the potential to improve registry utilization by trial sponsors and investigators, these measures do little good unless reviewers, journal editors, and educators utilize them. Recent evidence suggests that reviewers do not routinely use trials registries when assessing trial manuscripts [39]. In some cases there are good reasons to change study outcomes or other methodological details while a trial is ongoing, though it is critical that published manuscripts disclose and explain these changes. Other times, outcome changes may represent efforts by investigators to spin favorable conclusions from their data. In either case, reviewers and editors can and should use registry data to ensure that appropriate outcome changes are transparent and that inappropriate changes are prevented.

All but one of the studies included clinical trials that were initiated before 2005, which was when the ICMJE policy mandating prospective trial registration as a condition of publication in member journals went into effect. Registry utilization has increased dramatically since that time, but the quality of registry data remains inconsistent [22, 24]. The single study we examined that was limited to trials initiated after the ICMJE statement still found primary outcome inconsistencies in 15 of 55 (27 %) included trials [16]. Importantly, we did not find evidence of a consistent difference in registered and published outcomes according to trial funding source. As a result, any measures taken to improve the consistency of outcome reporting should target both industry-funded and non-industry-funded trials. As registry utilization continues to evolve, it is unclear whether outcome consistency will improve or whether inconsistencies will remain common. It is likely that this depends in part on the willingness of both journal editors and reviewers to demand that authors are held accountable to registered protocols when publishing results. Unfortunately, very few journals currently prioritize the assessment of registry entries during the peer review process [40].

Since 2008, in addition to facilitating the assessment of methodological consistency, Clinicaltrials.gov has included a results database that allows the registry to serves as a publicly available source of trial outcome data. While utilization of the results database on the part of investigators has been mixed, improved compliance with this feature has the potential to streamline comparisons between planned and completed outcome analyses [41]. Future surveillance studies will be needed to assess improvements in outcome consistency that might result from new policies, greater familiarity of investigators and sponsors with the registry process, and greater scrutiny of registries by editors and reviewers.

Our findings are consistent with those reported in a 2011 Cochrane Review of studies comparing trial protocols with published trial manuscripts [42]. This Cochrane Review identified three studies, all also included in our analysis, that compared registered primary outcomes to published outcomes, with observed primary outcome discrepancy rates between 18 % and 31 %. Additionally, the Cochrane Review identified three studies that compared the consistency between outcomes identified in study protocols and those in published reports, finding discrepancies in 33–67 % of included trials. As with our review, the presence of heterogeneous trial populations and methods prevented the Cochrane authors from performing a meta-analysis, though they observed that discrepancies between study protocols and published manuscripts commonly involved other important aspects of trial design in addition to outcome definitions, including eligibility criteria, sample size goals, planned subgroup analyses, and methods of analysis.

This systematic review has several limitations that should be considered when interpreting these results. First, studies involving outcome discrepancies are challenging to capture via a database search, because relevant studies are not limited to a single disease process or intervention, and many of the key words relevant to this topic are commonly included in unrelated abstracts (e.g., “bias,” “primary outcome,” “clinicaltrials.gov,” “randomized controlled trial”). We addressed this issue by using an intentionally broad search strategy, and by reviewing the reference lists of all included studies. Despite this, we may have missed some relevant publications. Second, the studies identified were heterogeneous with respect to both the characteristics of their included trials and the approaches they used to assess outcome consistency. This limits our ability to pool the data to provide a summary description of discrepancies in registered and published outcomes for trials across these studies. This heterogeneity results partly from the variable time points relative to trial initiation used by authors in defining the registered outcome, and is indicative of possible bias in these results at the level of individual studies. Third, we separately analyzed the subgroup of studies that reported reviewing the history of changes within each registry record in order to identify prospectively defined outcomes; this subgroup analysis may have been incomplete because it is possible that additional studies performed this task without reporting it. Further, several of the included studies had overlapping inclusion criteria, and as a result in some cases individual studies may have been included in more than one study. Additionally, the impact of publication bias on these results is unknown. Finally, many of the included studies noted that ambiguously defined outcomes among trial registries and manuscripts made it difficult to make judgments about whether outcome differences were present.

## Conclusions

Published randomized controlled trials commonly report primary outcomes that differ from the primary outcomes specified in clinical trials registries. These inconsistencies in published and reported outcomes are observed among trials across a broad range of clinical topics and a variety of funding sources. When assessing a trial’s methodological quality, editors, reviewers, and readers should routinely compare the published primary outcome to the primary outcome registered at the time of trial initiation. Further study, with attention to the outcome registered at the time of trial initiation, is needed to determine whether inconsistencies between registered and published outcomes remain common among recently initiated trials.

## Additional files

Additional file 1:
**Appendix A.** Electronic search strategies. (DOCX 495 kb) Additional file 2:
**Appendix B.** PRISMA 2009 checklist. (DOC 61 kb) Additional file 3:
**Appendix C.** Assessment of risk of study bias. (DOCX 24 kb)

## Abbreviations

ICMJE: International Committee of Medical Journal Editors
IQR: interquartile range
PRISMA: Preferred Reporting Items for Systematic Reviews and Meta-Analyses
WHO: World Health Organization
