# Supplementary material for: Comparison of registered and published outcomes in randomized controlled trials: a systematic review
Source: BMC Med. 2015 Nov 18;13:282. doi: 10.1186/s12916-015-0520-3 (PMC4650202; doi:10.1186/s12916-015-0520-3)
Supplement: Additional file 1: — Appendix A. Electronic search strategies. (DOCX 495 kb) [file 12916_2015_520_MOESM1_ESM.docx]

**APPENDIX A:** Electronic search strategies

MEDLINE via PubMed search:

#1. Registries/standards [MESH Terms]

#2. registration

#3. registry

#4. Clinicaltrials.gov

#5. ICTRP

#6. ISRCTN

#7. #1 OR #2 OR #3 OR #4 OR #5 OR #6

#8. primary outcome

#9. quality

#10. adequacy (adequa*)

#11. completeness

#12. consistency

#13. comparison (compar*)

#14. #9 OR #10 OR #11 OR #12 OR #13

#15. #7 AND #8 AND #14

#16. Case reports [pt]

#17. Randomized controlled trial [pt]

#18. Clinical trial [pt]

#19. #16 OR #17 OR #18

#20. #15 NOT #19

EMBASE search:

'registration'/exp OR registration OR 'registry'/exp OR registry OR clinicaltrials.gov OR ictrp OR isrctn AND 'primary outcome' AND (quality OR adequa* OR completeness OR consistency OR compar*) AND [embase]/lim NOT [medline]/lim AND [1-1-2004]/sd NOT [23-8-2014]/sd

CINAHL search

(registration OR registry OR clinicaltrials.gov OR ictrp OR isrctn) AND primary outcome AND (quality OR adequa* OR completeness OR consistency OR compar*)

Limits: Published Date 2001/01/01-2014/08/31, Exclude MEDLINE records
