# Supplementary material for: Comparison of registered and published outcomes in randomized controlled trials: a systematic review
Source: BMC Med. 2015 Nov 18;13:282. doi: 10.1186/s12916-015-0520-3 (PMC4650202; doi:10.1186/s12916-015-0520-3)
Supplement: Additional file 3: — Appendix C. Assessment of risk of study bias. (DOCX 24 kb) [file 12916_2015_520_MOESM3_ESM.docx]

**Appendix C:** Assessment of risk of study bias

1. Representativeness of the cohort
   1. Sample is truly representative of randomized controlled trials in general; includes a wide range of clinical topics, study sizes, and sponsors *
   2. Somewhat representative of the average randomized controlled trial (e.g. restricted to NIH-funded trials or cardiology trials) *
   3. Select group of trials (e.g. limited to trials on antihypertensive agents)
   4. No description of the study cohort, or unclear
2. Ascertainment of linkage between trial registry and publication
   1. Linkage established through registry identification number without hand registry search *
   2. Linkage established through combination of identification number and/or case by case comparison between registered and published study details (e.g. trial name, locations, funding source, planned sample size, etc) *
   3. No description or unclear:
3. Assessment of primary outcome agreement
   1. Independent assessment by multiple reviewers (i.e. by an investigator) *
   2. Self report by authors of included studies or assessment by single investigator
   3. No description or unclear
4. Limited to trials ongoing or started after July 2005
   1. Yes *
   2. No
   3. Unknown
5. Was registry history function used to assess registered primary outcomes prior to study completion?
   1. Yes *
   2. No

* indicates criteria associated with low risk of bias.

Reviews at low risk of bias are defined as having four or five out of five possible stars.

|  | Cohort is representative | Ascertainment of linkage between registry and publication | Assessment of primary outcome agreement | Limited to trials ongoing in or started after July 2005 | Used history function to assess prospectively registered outcomes |
| --- | --- | --- | --- | --- | --- |
| Anand V et al, 2014 | ***** | ***** | ***** | ***** | ***** |
| Bourgeois FT et al, 2010 |  | ***** | ***** |  |  |
| Chahal J, et al, 2012 | ***** | ***** | ***** |  |  |
| Ewart R et al, 2009 | ***** |  | ***** |  |  |
| Gandhi R et al, 2011 | ***** | ***** |  |  |  |
| Hannink G et al, 2013 | ***** | ***** | ***** |  | ***** |
| Hartung DM et al, 2014 | ***** | ***** | ***** |  |  |
| Huić M et al, 2011 | ***** | ***** |  |  | ***** |
| Hutfless S et al, 2013 |  |  |  |  | ***** |
| Jones CW et al, 2012 | ***** | ***** | ***** |  | ***** |
| Khan NA et al, 2012 |  | ***** | ***** |  |  |
| Killeen S et al, 2014 | ***** | ***** | ***** |  |  |
| Li XQ et al, 2013 | ***** | ***** | ***** |  |  |
| Liu JP et al, 2013 |  |  | ***** |  |  |
| Mathieu S et al, 2009 | ***** | ***** | ***** |  | ***** |
| Mathieu S et al, 2012 | ***** | ***** |  |  |  |
| Milette K et al, 2011 | ***** | ***** | ***** |  | ***** |
| Nankervis H et al, 2012 | AOttawa Scale for assessment of risk of study biasstered outcomest the level of individual reviews. reviews to determine outcome | ***** |  |  | ***** |
| Pinto RZ et al, 2013 | ***** | ***** | ***** |  |  |
| Rosenthal R et al, 2013 | ***** | ***** |  |  |  |
| Ross JS et al, 2009 | ***** | ***** |  |  |  |
| Smith HN et al, 2012 |  | ***** | ***** |  |  |
| Smith SM et al, 2013 |  |  | ***** |  |  |
| Vera-Badillo FE et al, 2013 |  | ***** |  |  |  |
| Walker KF et al, 2014 | ***** | ***** |  |  | ***** |
| Wildt S et al, 2011 | ***** | ***** | ***** |  |  |
| You B et al, 2012 | ***** | ***** | ***** |  |  |
